# Effect of vitamin D source and dietary cation–anion difference in peripartum dairy cows on calcium homeostasis and milk production

**DOI:** 10.1093/tas/txac010

**Published:** 2022-01-17

**Authors:** Matthew R Beck, Dakota Zapalac, James D Chapman, K P Zanzalari, Glenn A Holub, Scott S Bascom, Mark A Engstrom, R Ryan Reuter, Andrew P Foote

**Affiliations:** 1 Department of Animal & Food Sciences, Oklahoma State University, Stillwater, OK 74078, USA; 2 Phibro Animal Health, Teaneck, NJ 07666, USA; 3 DSM Nutritional Products, Inc., Parsippany, NJ 07054, USA

**Keywords:** dietary cation–anion difference, periparturient dairy cows, vitamin D

## Abstract

The objective of this experiment was to determine the effects of dietary vitamin D source on serum calcium (Ca), urinary Ca excretion, and milk production when fed in combination with a prepartum acidogenic negative dietary cation–anion difference (DCAD) diet. Nonlactating, pregnant multiparous cows (*n* = 15), balanced for breed (Holstein *n* = 9 and Jersey *n* = 6), and previous mature equivalent milk production, were assigned to one of three treatments (five cows/treatment), consisting of a control (PCH; positive DCAD, 8.9 mEq/100 g DM) and two negative DCAD diets (−15.4 mEq/100 g DM), one with vitamin D3 (cholecalciferol; NCH) and one with 25-hydroxyvitamin D3 (calcidiol; NCA; DSM nutritional products). The treatments were formulated to provide 1.95 mg/d of vitamin D and were fed 28 d prior to expected calving date. Delivery of vitamin D sources was accomplished by manufacture of a pellet and 2 kg of these pellets were individually fed simultaneously each day along with 2 kg of ground corn daily at 0800 hours. Negative DCAD treatments were formulated to provide 0.46 kg/d of Animate (Phibro Animal Health) and, if needed, additional Animate was top-dressed at each feeding to achieve a urine pH between 5.5 and 6.0 based on the previous day’s urine pH. Close-up cows had ad libitum access to chopped bermudagrass (*Cynodon dactylon* L.) hay and hay intake was measured using SmartFeed Pro systems (C-Lock Inc.; Rapid City, SD). Prepartum urine and serum samples were collected weekly and serum was collected 36, 48, and 72 h post-calving. Prepartum dry matter intake (DMI) as a percent of body weight was not (*P* = 0.66) affected by treatments. Cows fed NCH and NCA had greater (*P* = 0.02) prepartum serum Ca than PCH and tended to have greater urinary Ca excretions (*P* = 0.10). Average postpartum serum Ca (mg/dL) was greater (*P* = 0.05) for cows fed NCH (8.8) compared with PCH (7.8), whereas NCA (8.4) was numerically intermediate and not (*P >* 0.05) different from either of the other treatments. Postpartum DMI was not affected by treatment (*P* = 0.39). Daily milk yield (MY) (kg/d) was greatest (*P* < 0.01) for NCA (37.5) compared with the other treatments and NCH (34.1) was intermediate and greater than PCH (29.9). These results suggest that an acidogenic prepartum diet in combination with vitamin D was effective in maintaining peripartum serum Ca and the 25-hydroxy form of vitamin D improved MY compared with NCH in early lactation.

## INTRODUCTION

Improving calcium (Ca) homeostasis during the peripartum is one of the most important factors contributing to improved productivity and health in postpartum dairy cows. During the hours surrounding the period of calving, the Ca requirement of high producing dairy cows goes from approximately 20–50 g/d (Horst et al., 2005). There are a couple of prepartum feeding strategies that can improve the serum Ca status throughout the periparturient period and thereby reduce the negative health events and improve milk yield and reproductive performance (Chapinal et al., 2012). The most widely adopted feeding strategy to enhance the Ca status of periparturient dairy cows is the feeding of a prepartum acidogenic diet, also known as a negative dietary cation–anion difference (DCAD) diet. Additionally, feeding an acidogenic diet in combination with high dietary Ca has been shown to enhance postpartum dry matter intake (DMI), serum Ca concentrations, and milk yield (Leno et. al., 2017; Glosson et al., 2020) and improve certain markers of reproductive function (Ryan et al., 2020). By feeding an acidogenic diet, there is a resulting compensated metabolic acidosis, where blood and urine pH are reduced. This compensated metabolic acidosis is believed to increase the tissue responsiveness to parathyroid hormone, which directly induces mobilization of Ca from bones (Goff et al., 2014) and indirectly by increasing intestinal Ca absorption through increased formation of the active form of vitamin D in the kidney (Zhu and DeLuca, 2012). Glosson et al. (2020) demonstrated that feeding a negative DCAD diet increased blood ionized Ca through 24 h following calving and total blood Ca through 48 h post-calving. Another feeding strategy that has been demonstrated to improve Ca status in periparturient cows is the supplementation of vitamin D3. Vitamin D3, in its active form (1,25-hydroxyvitamin D3), is a hormone that plays a critical role in the regulation of blood Ca concentrations by increasing absorption of Ca from the gastrointestinal tract (Ramasamy, 2006). The regulation of vitamin D-25-hydroxylase expression and activity is not well understood but appears to be related to parathyroid hormone and 1,25-hydroxyvitamin D3 concentration, and potentially 25-hydroxyvitamin D3 (i.e., calcidiol; CA) concentration (Zhu and DeLuca, 2012). Parathyroid hormone increases the conversion of CA to 1,25-hydroxyvitamin D3 (i.e., the active form of vitamin D3), which has been suggested to explain why the benefits of vitamin D supplementation to Ca status is dependent on a negative DCAD diet (Wilkens et al., 2012). Ultimately, increasing the dietary concentration of Ca (Amundson et al., 2018), feeding a negative DCAD diet (Glosson et al., 2020), and providing additional vitamin D3 (Taylor et al., 2008 [CH and CA]; Wilkens et al., 2012 [CA]) in the diet are all well-established management tools to improve Ca status of periparturient dairy cows—resulting in less risk of negative health events such as milk fever (DeGroot et al., 2010).

There are benefits to feeding a negative DCAD diet in conjunction with the CA form of vitamin D3 in improving serum Ca status (Taylor et al., 2008; Wilkens et al., 2012). The objective of this experiment was to determine the effect of the form of vitamin D supplemented to cows in late gestation in combination with a negative DCAD diet on serum Ca concentrations, serum metabolites, and milk production. We hypothesized that feeding an acidogenic diet and high dietary Ca with two different sources of vitamin D would improve Ca homeostasis and productivity of periparturient cows compared with cows fed a negative control (positive DCAD and lower Ca content) and the effects would be enhanced when CA (calcidiol) is the vitamin D source rather than cholecalciferol (CH; D3).

## MATERIALS AND METHODS

### Animals and Treatments

All protocols were approved by the Oklahoma State University Institutional Animal Care and Use Committee (protocol AG-19-13). Pregnant, multiparous cows (*n* = 15) from the Ferguson Family Dairy at Oklahoma State University were selected for this study. Cows were blocked by breed (Holstein *n* = 9 and Jersey *n* = 6) and previous mature equivalent milk production and assigned to one of three treatments in a randomized complete block design. The average parity for the Holsteins was 3.1 and the Jerseys was 2.8. The average parity for each treatment was 2.8, 3.0, and 3.2 for the control, cholecalciferol, and calcidiol treatments, respectively. The mature equivalent milk yields were 11,078, 10,837, and 11,219 kg for the control, cholecalciferol, and calcidiol treatments, respectively. The sample size of *n* = 5 in each treatment was justified by a sample size calculation using serum Ca (mean = 9.22 mg/dL; SEM = 0.10) as reported by Wu et al. (2014). This calculation indicated that a sample size of *n* = 5 per treatment is required to find a 10% difference between treatments with an alpha = 0.05 and power = 90.

Prepartum treatments were formulated as pellets (Table 1). Treatments included a positive DCAD control with cholecalciferol (PCH) and two negative DCAD diets that differed by source of vitamin D: one included cholecalciferol (NCH) and the other included 25-hydroxyvitamin D_3_ (calcidiol; NCA; DSM Nutritional Products). All treatments were formulated to provide 1.95 mg/d of vitamin D3. Beginning 28 d prior to the expected calving date, the pellets (2 kg DM/d for each cow) were mixed with 2 kg of ground corn and fed to the cows in head-catch gates at 0800 hours each day. Cows receiving the negative DCAD treatments received additional Animate (90 g/d) to achieve and maintain the target urine pH of 5.5–6.0. Cows remained in the headlocks until the entire treatment and corn mixture was consumed, which typically spanned less than 30 min. The treatment diets were formulated assuming cows would consume 13.1 kg of hay DM/d. The formulated nutrient content of the treatment diets are presented in Table 2. Cows had ad libitum access to chopped bermudagrass (*Cynodon dactylon* L.) hay and feed intake was measured using SmartFeed Pro feeders mounted in trailers (C-Lock Inc.; Rapid City, South Dakota; Reuter et al., 2017).

**Table 1.** Ingredient composition of the treatment pellets fed beginning 28 d prior to expected calving and the lactation ration fed after calving

Ingredient, % of DM	Treatment pellets			Lactation ration
	PCH	NCH	NCA	
Wheat midds	38.93	23.49	23.49	-
Soy plus	31.49	29.84	29.84	-
AB-20	2.50	2.50	2.50	-
Distillers grains	16.00	-	-	5.94
Calcium carbonate	-	14.00	14.00	1.46
Dicalcium phosphate	-	1.00	1.00	0.15
Magnesium oxide	1.00	-	-	0.16
Soybean oil	-	1.00	1.00	-
Animate	-	17.99	17.99	-
Dry-rolled corn	-	-	-	32.17
Alfalfa hay	-	-	-	31.57
Whole cottonseed	-	-	-	9.01
Bermudagrass hay	-	-	-	6.23
Soybean hulls	-	-	-	4.83
SoyBest	-	-	-	4.24
Soybean meal	-	-	-	1.13
MegaLac	-	-	-	1.24
Sodium bicarbonate	-	-	-	1.09
Salt	-	-	-	0.55
Control premix∗	10.00	-	-	-
CH premix∗	-	10.00	-	-
CA premix∗	-	-	10.00	-
Lactation premix^†^	-	-	-	0.22
Flavoring agent	-	0.10	0.10	-
Rumensin 90	0.08	0.08	0.08	0.01

∗Composed of (DM basis) 11.77% calcium, 3.40% phosphorus, 15% magnesium, 2.28% sodium, 3.52% chloride, 4.07% sulfur, 565 ppm zinc, 471 ppm copper, 188 ppm manganese, 9.4 ppm cobalt, 28 ppm iodine, 357.0 KIU vitamin A/kg, 5.2 KIU vitamin E/kg, and 8.8 mg vitamin D/kg (as vitamin D3 for control premix and CH premix and as Rovimix [source of 25-hydroxyvitamin D3] for CA premix; DSM, Parsippany, NJ).

Composed of (DM basis) 2.043% calcium, 0.079% phosphorus, 0.045% magnesium, 0.232% potassium, 0.119% sodium, 5.490% chloride, 3.060% sulfur, 76,509 ppm zinc, 3,393 ppm copper, 68,122 ppm manganese, 968 ppm cobalt, 903 ppm iodine, 5.964 KIU vitamin A/kg, 29.9 mg vitamin D/kg, and 41,591 KIU vitamin E/kg.

**Table 2.** Nutrient composition of treatment close-up diets, bermudagrass hay fed to the dry cows, and the lactation ration

Component	Close-up diet∗			Bermudagrass hay	Lactation ration
	PCH	NCH^†^	NCA^†^		
DM, % as fed	90.73	90.83	90.83	91.51	61.04
CP, % DM	10.59	10.49	10.49	7.38	17.40
NDF, % DM	44.94	43.58	43.58	57.25	35.19
Starch, % DM	15.89	15.28	15.28	4.19	-
EE, % DM	2.81	2.59	2.59	1.81	-
Ca, % DM	0.58	1.56	1.56	0.35	1.09
P, % DM	0.40	0.39	0.39	0.23	0.44
Mg, % DM	0.43	0.48	0.48	0.18	0.29
K, % DM	1.08	1.02	1.02	1.13	1.76
Na, % DM	0.09	0.10	0.10	0.06	0.56
S, % DM	0.26	0.42	0.42	0.18	0.28
Cl, % DM	0.22	0.68	0.68	0.21	-
DCAD, mEq/100g	8.9	−15.4	−15.4	-	-

∗The close-up diets were formulated with cows expected to consume 13.1 kg of bermudagrass hay DM/d, 2 kg of ground corn DM/d, and 2 kg of the treatment pellet DM/d.

Treatments NCH and NCA received additional Animate (90 g/d) to achieve the targeted urine pH (5.5–6.0). The control and CH diet supplied 1.95 mg/d of vitamin D in the form of vitamin D3 and the CA diet supplied 1.95 mg/d of vitamin D3 in the form of 25-hydroxyvitamin D3.

After calving, cows were housed in a fresh cow pen at the Ferguson Family Dairy freestall barn for 2–3 d before being moved to a pen in the freestall barn equipped with an Insentec Feeding System (Insentec B.V., Marknesse, The Netherlands). The cows had ad libitum access to the lactation ration (Tables 1 and 2) and feed intake was recorded through 28 d in milk.

### Sample Collection and Measurements

While cows were consuming the treatment pellets and, in the head-catch gates, midstream urine samples were collected in a centrifuge tube beginning on day −28 and two consecutive days each week prior to calving. Urine pH was measured using litmus paper (Micro Essentials Laboratory Inc., Brooklyn, NY) and recorded. Samples were stored at −20 °C. Urine samples were later shipped to Phibro Animal Health Laboratories (Corvallis, OR) and analyzed for creatinine and Ca to estimate Ca excretion (Valadares et al., 1999).

Blood samples were collected via venipuncture of the coccygeal vein using Vacutainers with no additives (Becton, Dickinson and Company, Franklin Lakes, NJ) on d −60, −45, −28, −14, and −7 before the expected calving date and 36, 48, and 72 h after calving. Samples were allowed to clot for 30 min and then centrifuged at 3,000 × *g* at 4 °C for 25 min. Serum was then aspirated and stored at −20 °C. A subsample of serum was analyzed for minerals by the Michigan State University Veterinary Diagnostic Laboratory (Lansing, MI). Serum was also analyzed for urea nitrogen using the protocol described by Marsh et al. (1957) and nonesterified fatty acids using a modified protocol of the NEFA-HR (2) kit (Wako Pure Chemical Corporation, Osaka, Japan). Cow body weights were collected on a walk-across scale (Gallagher Animal Management, Riverside, MO) and body condition score was recorded by the same scorer at −60, −28, −14, and −7 d prior to the expected calving date.

Following calving, cows were milked twice daily, and milk weights were recorded (DeLaval, Kansas City, MO) for the first 28 d of lactation. Milk samples were collected at both milkings for two consecutive days of the first 2 wk of lactation. Samples were analyzed for fat, protein, somatic cell count, and milk urea nitrogen (Dairy One, Ithaca, NY). Milk component yield was calculated as the product of milk produced and component percentage. Fat corrected milk (FCM) yield was calculated as: 3.5% FCM kg/d = (0.432 × milk kg/d) + (16.216 × fat kg/d) (Dairy Records Management Systems, 2014). Energy corrected milk (ECM) production was calculated according to Tyrrell and Reid (1965) using the equation: ECM kg/d = (0.327 × milk kg/d) + (12.95 × fat kg/d) + (7.65 × protein kg/d).

### Statistical Analysis

All data were analyzed using SAS 9.4 (SAS Institute Inc., Cary, NC). Data were analyzed using the mixed procedure including treatment, time, and the interaction as fixed effects and breed as a covariate. Time was treated as a repeated measure with animal as the subject. The covariance structure yielding the lowest Akaike Information Criterion was used. Covariance structures tested included the following and the variable it was used for is reported in parentheses: autoregressive 1 (prepartum BCS, milk protein content, prepartum and postpartum blood potassium, postpartum blood calcium, postpartum blood sodium, and postpartum BUN), heterogeneous autoregressive 1 (prepartum NEFA, milk production, milk fat percentage, milk protein yield, FCM, MUN, prepartum blood phosphorus, and postpartum blood magnesium), compound symmetry (all other variables), heterogenous compound symmetry (DMI as percent of BW, milk fat yield, ECM, prepartum blood magnesium, prepartum blood sodium, and prepartum blood chloride), ante-dependence 1 (prepartum blood calcium, prepartum BUN, and postpartum NEFA), and unstructured (postpartum blood phosphorus). All variables were evaluated for normality by visually inspecting the residual plots from the mixed model analysis. Ca excretion and somatic cell counts were found to be non-normal and were log transformed to achieve normality. *P*-values ≤ 0.05 were considered significant.

## RESULTS

One Holstein cow in the NCA group developed mastitis shortly after calving and was removed from the study. It is unclear if the mastitis was related to the prepartum treatment. The prepartum data of the cows were included in the analyses, but no postpartum data was used. No other clinical health events were observed in any cows.

### Effects of Treatments on Prepartum Variables

**Figure 1.**
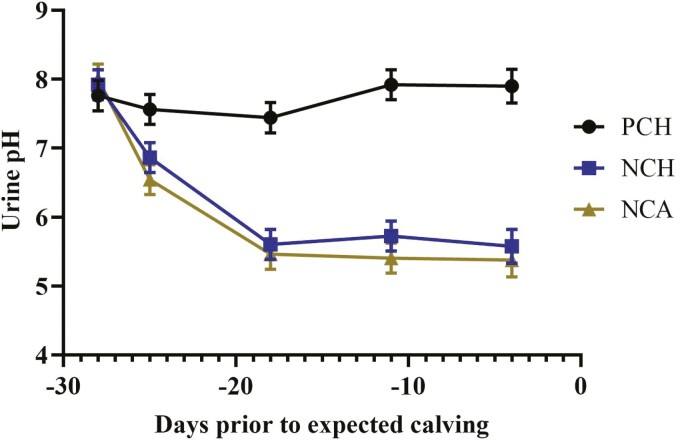
Effect of close-up diet on urine pH prior to calving.

Prepartum hay intake did not differ between treatment when expressed as kg DM/d (*P* = 0.86; Table 3) or as a percentage of BW (*P* = 0.66). Holsteins tended to consume more hay DM/d than Jersey cows (8.32 vs. 5.59 kg/d, respectively; *P* = 0.08) as well as on a BW basis (1.46% vs. 0.87% of BW for Holsteins and Jerseys, respectively; *P* = 0.06). There was no effect of treatment (*P* = 0.46) or time (*P* = 0.30) on BCS in the dry cows. Cows consumed the entirety of the treatment pellets and ground corn, usually within 30 min. During the first 3 d of offering the treatment pellets, it was observed that the cows receiving the NCH and NCA treatments consumed the pellets more slowly than the control cows; however, NCH and NCA fed cows did consume the entire daily ration. Urine pH of the cows fed the prepartum acidogenic diets were lower (*P* < 0.001) than the urine pH of control cows. There was a treatment × time interaction (Figure 1; *P* < 0.001) for urine pH, but there was essentially no difference between the NCH and NCA treatments.

**Table 3.** Effect of close-up diet on prepartum feed intake, urine pH, and serum minerals and metabolites

Item	Treatment			SEM	*P*-values		
	PCH	NCH	NCA		Treatment	Time	Treatment × time
Prepartum hay intake, kg DM/d	6.87	7.42	6.56	1.298	0.86	0.002	0.84
DMI, % of BW	1.21	1.29	0.99	0.259	0.66	0.003	0.94
Prepartum BW, kg	587	643	653	36.9	0.41	0.29	0.17
Prepartum BCS	3.50	3.52	3.66	0.122	0.46	0.30	0.86
Urine pH	7.72^a^	6.34^b^	6.16^b^	0.100	<0.001	<0.001	<0.001
Serum							
Calcium, mg/dL	8.81^b^	9.30^a^	9.18^a^	0.105	0.008	0.20	0.29
Phosphorus∗, mg/dL	7.16	6.81	6.34	0.256	0.09	<0.001	0.011∗
Magnesium, mg/dL	2.04	2.16	2.28	0.111	0.33	0.83	0.65
Sodium, mmol/L	138.6	139.0	139.6	0.66	0.53	0.001	0.73
Potassium^†^, mmol/L	4.59	4.79	4.67	0.102	0.38	0.35	0.03^†^
Chloride, mmol/L	101.2	102.0	103.6	0.87	0.16	<0.001	0.64
NEFA, µEq/L	202.4^b^	304.3^ab^	450.8^a^	63.25	0.04	0.23	0.99
BUN, mg/dL	13.14	13.36	16.31	1.275	0.20	0.72	0.35
Log calcium excretion	0.091	0.694	0.616	0.191	0.08	0.08	0.57
Calcium excretion, g/d	1.23	4.94	4.13	-	-	-	-

∗See Figure 4 for the interactive means for serum phosphorus.

See Figure 5 for the interactive means for serum potassium.

**Figure 2.**
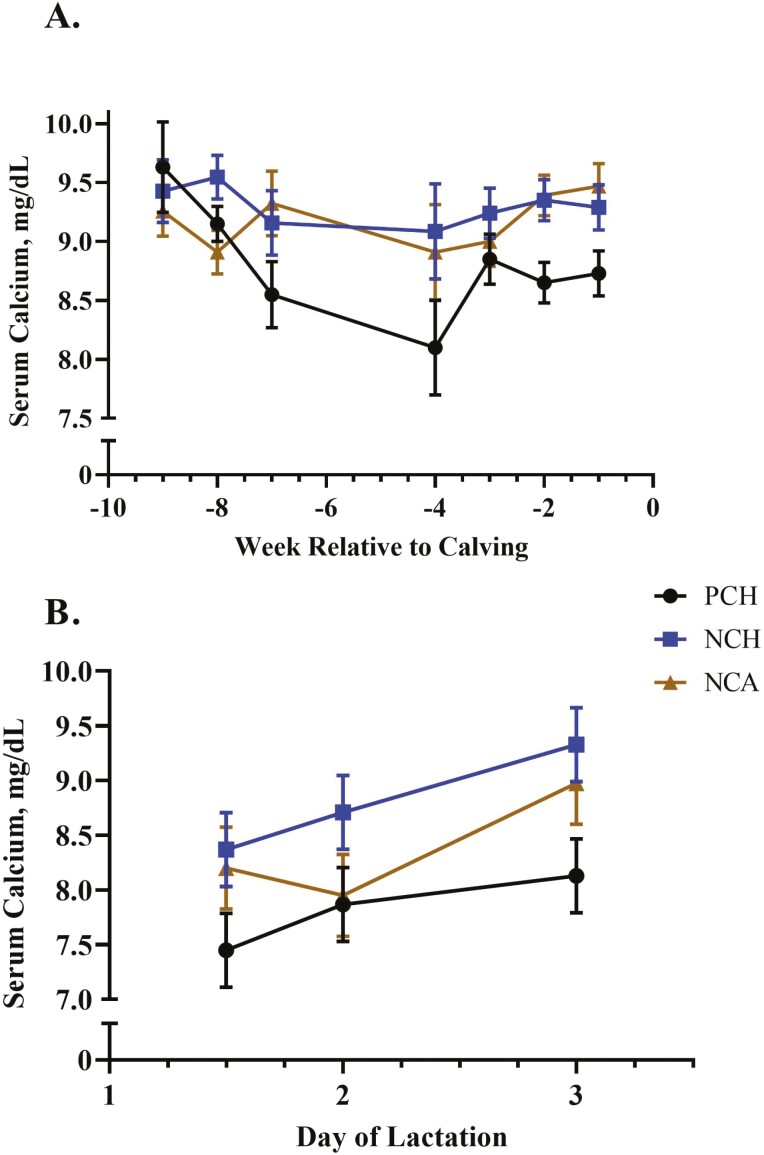
Effect of close-up diet on prepartum (A) and postpartum (B) serum calcium concentration. Treatment × time interaction was not significant for pre- or postpartum periods (*P* ≤ 0.29). Control cows receiving a positive DCAD (PCH) diet had lower serum calcium prior to calving compared with the other two treatments. Additionally, the negative DCAD diet with calcidiol (NCA) treatment had greater (*P* < 0.05) serum calcium than the PCH cows, while negative DCAD with cholecalciferol (NCH) cows were not different (*P* > 0.05) from the other two treatments during the postpartum period.

**Figure 3.**
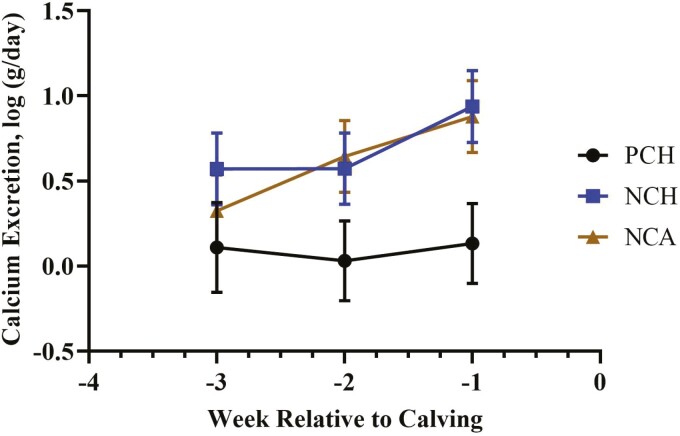
Effect of close-up diet on calcium excretion prepartum. The negative DCAD with cholecalciferol (NCH) and the negative DCAD diet with calcidiol (NCA) cows tended (*P* = 0.08) to have greater calcium excretions than the positive DCAD with calcidiol control (PCH) cows and calcium excretion was not significantly (*P* = 0.08) influenced by day.

**Figure 4.**
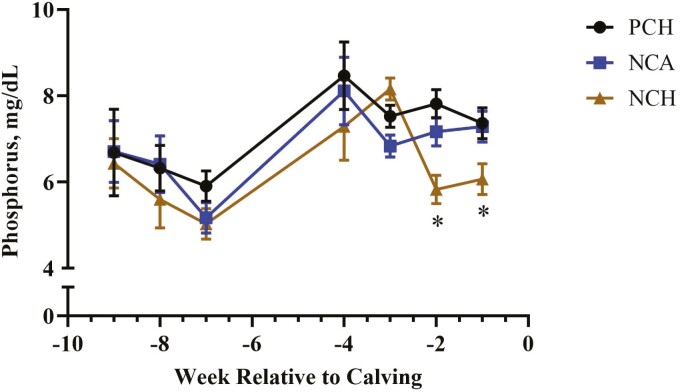
Effect of close-up diet on serum phosphorus concentrations prepartum. Treatment × time, *P* = 0.011. The negative DCAD diet with calcidiol (NCA)-treated cows differed on weeks −2 and −1 relative to calving.

**Figure 5.**
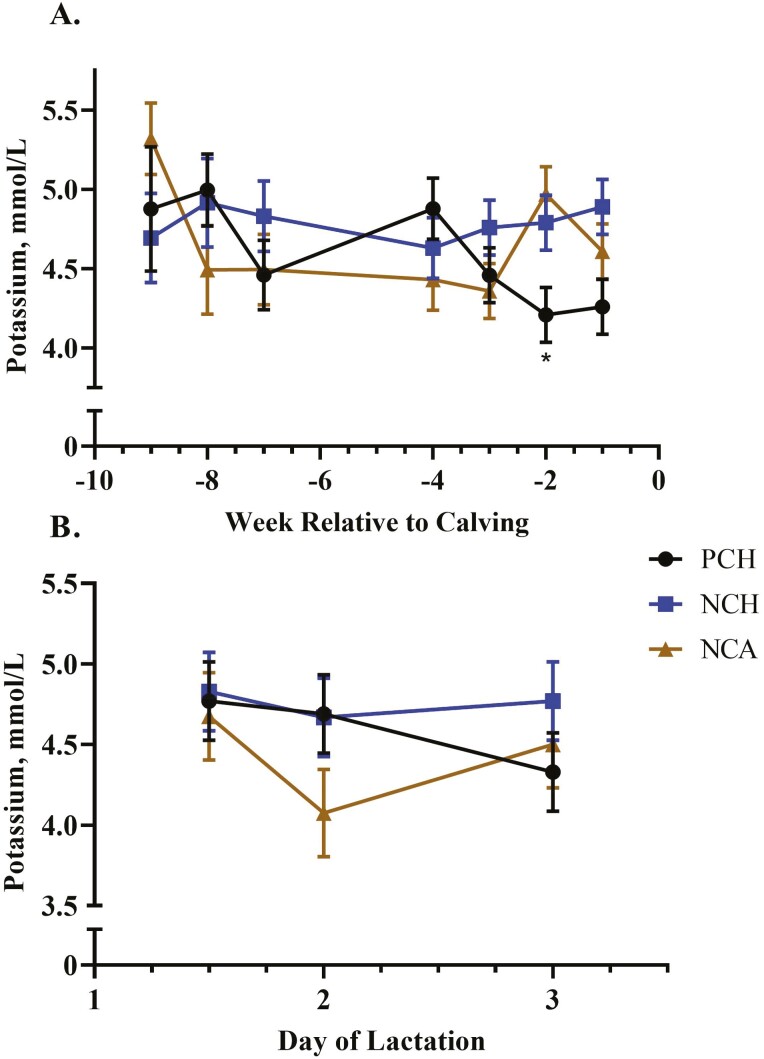
Effect of close-up diet on prepartum (A) and postpartum (B) serum potassium concentration. Treatment × time interaction, *P* ≤ 0.03. Control cow receiving a positive DCAD (PCH) diet had lower serum potassium on week −2 relative to calving compared with the other two treatments. The negative DCAD with cholecalciferol (NCH)-treated cows had consistent potassium concentrations in the first 3 d after calving, while negative DCAD diet with calcidiol (NCA)-treated cows displayed a decrease on day 2, and a subsequent increase on day 3 after calving.

Serum Ca was greater (*P* = 0.008) for cows fed either of the two negative DCAD diets but did not differ between the two sources of vitamin D (Table 3 and Figure 2A). Calcium excretion tended (*P* = 0.08; Table 3 and Figure 3) to be affected by treatment, with cows fed the negative DCAD diets excreting more Ca through urine than control fed cows. There was an interaction of treatment × time (*P* = 0.011; Figure 4) for serum phosphorus. Serum phosphorus increased in weeks −4 and −5 compared with the far-off samples (weeks −7 and −8), but concentrations dropped in the NCA-treated cows in weeks −2 and −1. There was a treatment × time interaction (*P* = 0.03; Figure 5A) for serum potassium, which was due to a decrease in serum potassium of the control-treated cows in week −2 compared with the NCH and NCA treatments. There was also a time effect for serum sodium (*P* < 0.01) and chloride (*P* < 0.01). While differences were not dramatically different between the treatments, sodium concentrations were lowest at weeks −8 and −7 and greatest at week −1. Similarly, chloride concentrations were lowest during weeks −8 to −3 and then increased during weeks −2 and −1. Serum NEFA were greatest for the NCA-treated cows (*P* = 0.04), lowest for the control cows, and intermediate for the NCH cows. Prepartum BUN was not affected by treatment (*P* = 0.20) or time (*P* = 0.72).

### Effect of Treatments on Postpartum Variables

Prepartum treatment had no effect on postpartum DMI of the lactation ration (*P* = 0.39; Table 4). Milk production (kg/d) was approximately 25% greater from cows receiving the NCA treatment compared with control cows (*P* < 0.01) and approximately 10% greater than cows fed the NCH diet. Additionally, the NCH-treated cows produced about 13.7% more milk than control cows. The NCA treatment group had decreased milk fat (*P =* 0.01) and protein (*P* = 0.02) percentages compared with the control cows. However, yield (kg/d) of milk fat (*P* = 0.52) and protein (*P* = 0.11) did not differ between treatments. The FCM (*P* = 0.66) and ECM production (*P* = 0.84) were not different between the treatments. Somatic cell counts (*P* = 0.94) and MUN (*P* = 0.92) were not affected by close-up diet treatment.

**Table 4.** Effect of close-up diet on postpartum production measurements and serum minerals and metabolites

Item	Treatment			SEM	*P*-values		
	PCH	NCH	NCA		Treatment	Time	Treatment × time
DMI, kg DM/d	11.27	10.15	10.62	0.615	0.39	<0.001	0.75
DMI, % of BW	1.91	1.62	1.64	0.125	0.19	<0.001	0.58
Postpartum BW, kg	556	609	599	36.8	0.51	<0.001	0.13
Postpartum BCS	2.98	3.28	3.17	0.119	0.18	<0.001	0.96
Milk production, kg/d	29.93^c^	34.05^b^	37.45^a^	1.200	< 0.001	<0.001	0.96
3.5% FCM, kg/d	40.56	42.38	41.25	1.593	0.66	<0.001	0.94
Energy corrected milk, kg/d	40.79	42.61	42.31	2.600	0.84	<0.001	0.88
Milk fat, %	5.86^a^	5.21^ab^	4.14^b^	0.334	0.01	0.24	0.89
Milk fat yield, kg/d	1.71	1.71	1.54	0.120	0.52	<0.001	0.60
Milk protein, %	4.17^a^	3.83^ab^	3.70^b^	0.105	0.02	<0.001	0.19
Milk protein yield, kg/d	1.16	1.22	1.33	0.058	0.11	<0.001	0.92
Log somatic cell	2.05	2.11	2.15	0.22	0.94	0.08	0.32
SCC	112	129	141	-	-	-	-
Milk urea nitrogen	8.41	8.86	8.18	1.260	0.92	0.10	0.36
Serum							
Calcium, mg/dL	7.82^b^	8.80^a^	8.38^ab^	0.289	0.05	0.009	0.50
Phosphorus, mg/dL	5.60	5.94	5.94	0.469	0.81	0.43	0.87
Magnesium, mg/dL	1.75	1.97	2.00	0.145	0.40	0.06	0.13
Sodium, mmol/L	144.2	143.1	143.4	0.66	0.44	0.004	0.88
Potassium∗, mmol/L	4.60	4.76	4.42	0.244	0.60	0.03	0.02∗
Chloride, mmol/L	102.7	101.4	102.2	1.14	0.69	0.14	0.19
NEFA^†^, µEq/L	129.1	355.9	457.1	49.36	0.002	0.10	0.03^†^
BUN, mg/dL	12.4	13.6	14.0	1.60	0.76	0.18	0.37

∗See Figure 3 for the interactive means for serum potassium.

See Figure 4 for the interactive means for serum NEFA.

**Figure 6.**
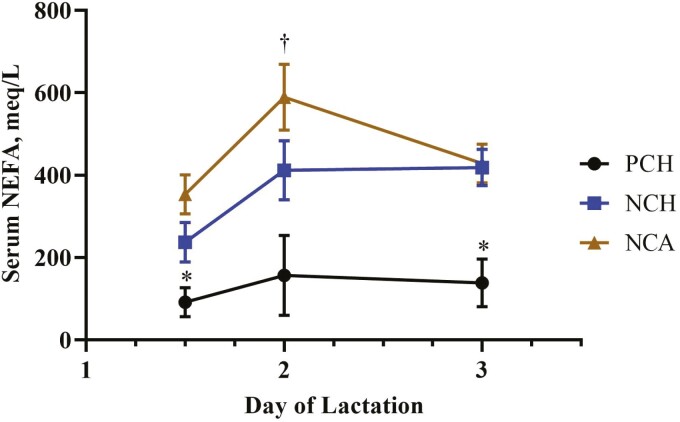
Effect of close-up diet on postpartum serum nonesterified fatty acid (NEFA) concentration. Treatment × time, *P* = 0.03. Control cows receiving a positive DCAD (PCH) diet had NEFA concentrations that were lower (*P* = 0.042) than the negative DCAD with cholecalciferol (NCH) and negative DCAD diet with calcidiol (NCA)-treated cows at 36 h and 3 d after calving (*P* = 0.004) and only differed (*P* = 0.007) from the NCA-treated cows on day 2 of lactation.

Post-calving, there was no interaction between time and treatment for serum Ca (*P* = 0.50), but it was greater in NCH-treated cows compared with control cows (*P* = 0.05), with NCA cows intermediate to the other two treatments. For all cows, serum Ca increased over the first 72 h after calving (*P* < 0.01; Figure 2B). There was an interaction of treatment × time for serum potassium (*P* = 0.02; Figure 6B) due to a decrease in potassium in NCA-treated cows on day 2 post-calving followed by an increase on day 3. Serum NEFA concentrations were lowest for the PCH cows on all sampling time-points post-calving (*P* = 0.03; Figure 6) but did not differ between cows fed NCH and NCA treatments on day 2.

## DISCUSSION

We hypothesized that 1) a negative DCAD prepartum diet and 2) the source of vitamin D3 (calcidiol vs. cholecalciferol) would improve Ca status and productivity of periparturient cows. Our results support these hypotheses as the NCH and NCA cows had greater milk production and prepartum serum Ca levels compared with CON cows. Further, NCH fed cows had significantly greater and NCA fed cows tended to have greater postpartum serum Ca levels compared with the PCH fed cows. In addition, cows fed CA had greater milk production than NCH fed cows. However, there were no observed differences in serum Ca concentration between the cows offered the two sources of vitamin D.

Cows fed NCA and NCH treatments had greater (6.3%) serum Ca prior to calving compared with the control cows. Additionally, NCH cows had significant (12.5% greater) and NCA cows tended to have greater serum Ca concentration compared with control cows post-calving. It is hard to determine the exact cause for this difference, as the NCA and NCH treatment groups were fed a negative DCAD diet, with 2.7-times greater dietary Ca concentration and vitamin D additions, in contrast with the control close-up diet, which contained only NCH. However, Kronqvist et al. (2011) determined no benefit to blood Ca around parturition when cows were fed dietary treatments ranging from 0.5% to 1.4% Ca, which may indicate that the differences between control and the other two treatments were a result of a negative DCAD diet. Serum calcium data presented here indicate that adding vitamin D to a positive DCAD diet (PCH) does not improve Ca status of periparturient cows, as these cows displayed serum Ca concentrations indicative of subclinical hypocalcemia (discussed later). A negative DCAD diet and addition of vitamin D have been shown as beneficial management protocols to improve the blood Ca status of the periparturient dairy cow (Wilkens et al., 2012). The level of anion supplementation was adequate to achieve the level of compensated metabolic acidosis to be beneficial for improved blood Ca levels, as evidenced by the urine pH measured prior to calving. While we are unable to definitively isolate what effect DCAD, Ca content of the diet, and vitamin D addition had on serum Ca levels in the current experiment, Leno et al. (2017) showed that equal dietary Ca in diets with increasingly negative DCAD had little effect on prepartum blood Ca, but did improve postpartum blood Ca, postpartum DMI, and milk yield. These results combined with previous research (Wilkens et al., 2012; Leno et al., 2017) suggest that feeding close-up dairy cows a negative DCAD diet with greater Ca content and additional vitamin D sources provided a means to improve blood Ca levels in periparturient dairy cows, thereby better meeting the additional Ca requirements caused by the onset of lactation and attenuating transition disorders caused by hypocalcemia.

In the current experiment, serum Ca concentration was not significantly different between the NCA and NCH source of vitamin D addition during both pre- and postpartum periods. This agrees with Taylor et al. (2008) and Rodney et al. (2018) who also determined no benefit to blood Ca levels between cows fed NCA and NCH. Addition of vitamin D as NCA has been shown to increase blood levels of CA, above physiological limitations which exist in the conversion of CH to CA. 25-hydroxyvitamin D3 is further converted to the active form of vitamin D (i.e., 1,25-dihydroxyvitamin D3) which is involved in increasing absorption of Ca from the gastrointestinal tract (Ramasamy, 2006). Supplementing CA has been associated with increases in blood concentration of 1,25-dihydroxyvitamin D3 prepartum compared with CH supplemented cows (Rodney et al., 2018). If CA supplementation increases 1,25-dihydroxyvitamin D3 concentrations, then it may be expected for CA to increase serum Ca levels than when CH is supplemented. In both the current experiment and Rodney et al. (2018), NCA increased milk production compared with inclusion of CH was the vitamin D source. If NCA induces greater Ca milk excretion than NCH, then this may explain why NCA supplemented cows did not have greater blood Ca than NCH supplemented cows in the current experiment.

Cows supplemented with negative DCAD and CH or CA in close-up rations had 13.8% and 25.1% greater milk production (kg/d) compared with control cows, respectively. This may be explained by the improved Ca status for the NCH cows and the tendency for improved Ca status for NCA cows compared with control cows, respectively. A serum Ca below 2 mmol/L (8.02 mg/dL) has been suggested as the threshold for subclinical hypocalcemia in periparturient dairy cows (Reinhardt et al., 2011), while other reports use 2.125 mmol/L (8.5 mg/dL) as the threshold for subclinical hypocalcemia (Chapinal et al., 2012; Martinez et al., 2018). The average PCH cow was below the lower threshold, whereas NCA cows were below the 8.5 mg/dL threshold until 3 d post-calving and NCH cows were above 8.5 mg/dL by 2-d postpartum. This may indicate that the control treatment group had greater prevalence of subclinical hypocalcemia than the NCH and NCA cows. Although still above the thresholds which induces milk fever (i.e., periparturient paresis), subclinical hypocalcemia has been shown to negatively influence milk production (Seely et al., 2021). Thus, these results may indicate that control cows had greater prevalence of subclinical hypocalcemia, thereby potentially explaining the greater milk production for NCH and NCA measured in the current experiment. This interpretation of the current results is supported by Santos et al. (2019) who determined that acidogenic diets increase milk production using a meta-analysis approach. It is unlikely that the cows receiving the NCA treatment were negatively impacted by the slightly decreased blood Ca concentration in the first 2 d of lactation.

The NCA cows had greater (10%) milk production than NCH cows. This is in agreement with Martinez et al. (2018) who determined that cows provided vitamin D through CA had (11.8%) greater milk production than when provided vitamin D through CH. However, Martinez et al. (2018) reported an increase in FCM and ECM for CA-fed cows compared with CH cows, whereas we did not detect this in the current experiment, albeit this discrepancy may be related to the limited number of animals used in the current experiment. Feeding vitamin D as CA has resulted in greater blood concentration of CA compared with when vitamin D is fed as CH, even though CH is converted to CA by vitamin D-25-hydroxylases in the liver. Vitamin D addition as CH results in marked increases of blood CH levels, but there is evidently some physiological threshold in the conversion of CH to CA (90–100 ng/mL in the plasma; Rodney et al., 2018). Therefore, supplementing CA directly may result in blood levels of CA above that physiological threshold. Some research has suggested that CA may have direct effects on mammary cells, thereby potentially explaining the increased milk production (Martinez et al., 2018). Thus, the NCA supplemented cows likely had greater blood concentrations of CA compared with NCH supplemented cows and the greater CA concentration may have direct effects on mammary cells, thereby possibly explaining the greater milk production for NCA cows than NCH cows in the current experiment.

One of the current results that was not expected was the NCA and NCH cows had greater serum NEFA concentrations in the pre- and postpartum phases compared with the control cows. There is a known relationship between energy and calcium metabolism (Lean et al., 2014). When measured on the same day, there is a negative correlation between blood Ca and NEFA concentrations (Lean et al., 2014). Further, Reinhardt et al. (2011) compared serum samples from 1,462 cows within 48 h of parturition, which were analyzed for serum Ca and NEFA. They concluded that cows with subclinical hypocalcemia (<2 mmol Ca/L [i.e., <8.02 mg Ca/dL]) had significantly greater serum NEFA compared with cows that did not have subclinical hypocalcemia (>2 mmol Ca/L). While not confirming the conclusion of Reinhardt et al. (2011), several experiments which have explored the effects of dietary DCAD and addition of vitamin D reported no effects on NEFA (Moore et al., 2000; DeGroot et al., 2010; Martinez et al., 2018). Changes in BW and body condition postpartum were not affected by treatment and do not explain the differences observed. Nonesterified fatty acids have been shown to have a positive relationship with milk production and this relationship may explain why the NCA and NCH cows in current experiment had greater NEFA concentrations postpartum in the current experiment. However, milk production would not explain why NCA and NCH cows had greater NEFA concentrations prepartum. While there was no time or treatment × time effects on prepartum NEFA concentrations, serum NEFA was elevated in NCA and NCH cows in the dry period prior to initiating the treatments. It is possible that these results related to NEFA concentrations are not related to DCAD or vitamin D treatments. Further research is recommended to determine the cause.

## CONCLUSION

The current experiment determined that feeding close-up dairy cows a negative DCAD diet with supplemental vitamin D increased serum Ca concentration prior to calving. The NCA and NCH cows had greater milk production compared with the control cows, which is potentially due to control having serum Ca below the suggested threshold for subclinical hypocalcemia. However, component yield for fat- or ECM were improved by the NCA and NCH treatments. Additionally, the NCA cows had greater milk production compared with NCH cows. The effect of NCA on milk production has been suggested to be a result of a direct biological effects on mammary cells. Based on these results, a negative DCAD diet with additional vitamin D enhances the Ca status of the periparturient dairy cow. Finally, providing NCA may increase milk production further than when vitamin D is provided as NCH.
